# Ugandenial A, a New Drimane-type Sesquiterpenoid from *Warburgia ugandensis*

**DOI:** 10.3390/molecules14103844

**Published:** 2009-09-28

**Authors:** Min Xu, Marc Litaudon, Sabrina Krief, Marie-Thérèse Martin, John Kasenene, Bernard Kiremire, Vincent Dumontet, Françoise Guéritte

**Affiliations:** 1 Kunming Institute of Botany, Chinese Academy of Sciences, Heilongtan, Kunming 650204, China; E-Mail: xumin@mail.kib.ac.cn (M.X.); 2 Institut de Chimie des Substances Naturelles, UPR2301, CNRS, 91198 Gif-sur-Yvette cedex, France; E-Mails: mtm@icsn.cnrs-gif.fr (M-T.M.); vincent.dumontet@icsn.cnrs-gif.fr (V.D.); francoise.gueritte@icsn.cnrs-gif.fr (F.G.); 3 UMR 7206-USM 104 Eco-anthropologie et ethnobiologie, MNHN, 75231 Paris cedex, France; E-Mail: krief@mnhn.fr (S.K.); 4 Department of Botany, Makerere University, Box 7062, Kampala, Uganda; E-Mail: botany@botany.mak.ac.ug (J.K.); 5 Chemistry Department, Makerere University, Box 7062, Kampala, Uganda; E-Mail: kiremire@chemistry.mak.ac.ug (B.K.)

**Keywords:** *Warburgia ugandensis*, drimane-type sesquiterpenoid, ugandenial, cytotoxicity

## Abstract

One new drimane-type sesquiterpenoid, named ugandenial A (**1**), was isolated from the ethyl acetate extract of the bark of *Warburgia ugandensis* (Canellaceae) together with eight known drimane-type sesquiterpenoids: 11α-hydroxycinnamosmolide (**2**), warburganal (**3**), polygodial (**4**), mukaadial (**5**), dendocarbin A (**6**), 9α-hydroxycinnamolide (**7**), dendocarbin L (**8**) and dendocarbin M (**9**). Their structures were established by detailed spectroscopic analysis. In addition a keto-enol equilibrium was demonstrated for compound **1** through a detailed NMR analysis run in CD_2_Cl_2_ at 190 K. Cytotoxicity of the isolated compounds against KB cells was evaluated.

## Introduction

In order to better understand the potential self-medicative behavior of chimpanzees, one of us (SK) has investigated since 2000 the bioactivities of plant species eaten by wild chimpanzees in Kibale National Park in Uganda. Among 300 samples collected so far and screened for different bioactivities, the EtOAc extract of *Warburgia ugandensis* bark exhibited potent cytotoxic activity on KB cell line (99 % and 64% inhibition at 10 and 1 μg/mL, respectively). This evergreen straight tree may reach 30 m in height. The leaves are short-stalked, simple, alternate, dotted with glands and lacking stipules. Berries, purple when dried contain several heart-shaped seeds in a cream-green pulp. All parts of the tree are known by local people to have a hot aromatic taste and the spicy leaves are used as a chili substitute [1]. Roots and bark are used in traditional medicine especially to treat worms, pains, cough, colds and malaria [2]. However, traditional healers are cautious and use only small quantities as it may cause violent vomiting [1,3].

In previous phytochemical investigations of *W. ugandensis*, a large number of drimane-type sesquiterpenoids have been isolated from the heart wood, like ugandensolide, ugandesidial, warburgin and warburgiadione [4] and from the stem bark [5,6,7], like muzigadiolide, deacetylugandensolide, cinnamolide, mukaadial, ugandensidial, muzigadial, waburganal and others, while flavonol glycosides [8] and monoterpenes [5] were characterized from *W. ugandensis* leaves. The chemical investigation of the EtOAc bark extract was undertaken since a strong cytotoxicity was found against KB cells. Bioassay-guided purification of this extract by repeated column chromatography, semi-preparative HPLC and preparative TLC led to the isolation of one new compound **1**, together with eight known drimane-type sesquiterpenes: 9α,11α-dihydroxy,6β-acetyl-cinnamolide (**2**), warburganal (**3**), polygodial (**4**), mukaadial (**5**), 9α-hydroxycinnamolide (**7**), and dendocarbins A, L and M (**6**, **8** and **9**, respectively) reported for the first time in this species. Their structures were established by detailed spectroscopic analysis. In addition a keto-enol equilibrium was demonstrated for compound **1** through a detailed NMR analysis run in CD_2_Cl_2_ at 190 K. Cytotoxicity of the isolated compounds against a KB cell line was also evaluated. 

## Results and Discussion

The EtOAc bark extract (3 g) of *W. ugandensis* Sprague was subjected to column chromatography on silica gel, semi-preparative HPLC, and preparative TLC to afford one new compound **1**, in addition with eight known ones **2**-**9**, which were identified by comparison of the spectral data with the reported literature values.

Compound **1** was obtained as a white amorphous powder. Its molecular formula was determined as C_15_H_22_O_4_ from the [M+Na]^+^ pseudomolecular ion peak at *m/z* 289.1427 (calc. 289.1416) in the HRESIMS. The IR spectrum of compound **1** revealed a broad absorption at 3,440 cm^-1^, characteristic of an OH stretch, and a strong absorption at 1,745 cm^-1^, characteristic of an ester or lactone carbonyl stretch. The ^1^H- and ^13^C-NMR data (Table 1) of **1** were closely comparable to those of 11α-hydroxycinnamosmolide isolated from *Warburgia salutaris* [9].

**Figure 1:**
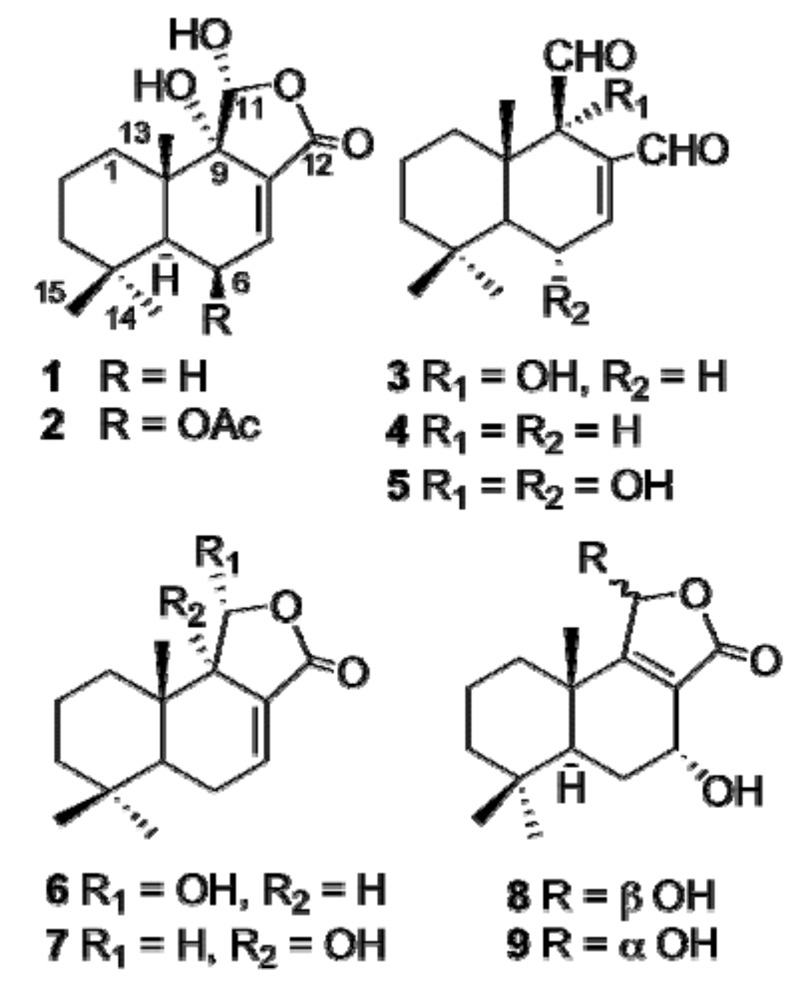
Structures of compounds **1**-**9**.

**Figure 2:**
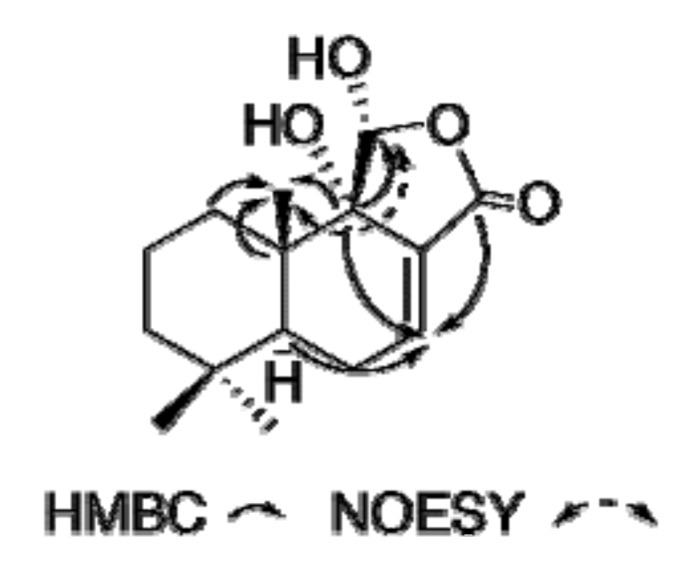
Key HMBC and NOESY interactions for compound **1**.

In the HMBC spectrum of **1** in CD_2_Cl_2_ at 190 K (Figure 2), correlations from the methine at 41.1 (C-5), the quaternary carbon at 75.7 (C-9) and the carbonyl at 168.7 (C-12) to the olefinic proton at 6.98 (*brs*, H-7) confirmed the presence of a conjugated γ-lactone moiety. On the basis of the above spectral data, as well as HMQC, ^1^H-^1^H COSY analyses and other HMBC correlations, compound **1** is a typical drimane sesquiterpene possessing a C-ring. However, due to a keto-enol equilibrium the signals for the H-11 and C-11 were not visible, either in CDCl_3_, CD_3_OD, CD_2_Cl_2_ or DMF at room temperature. A NMR study at 190 K in CD_2_Cl_2_ showed that a doublet at δ_H_ 5.66 (*J* = 10 Hz), which was assigned to H-11 is observable in the ^1^H-NMR spectrum; the doublet give a sharp singlet after irradiation of the OH-11 at δ_H_ 5.90. Moreover, at 190 K, a cross peak between H-11 and C-9 at δ_C_ 75.7 in the HMBC spectrum, and the HMQC correlation indicating the chemical shift of C-11 was 98.5 confirmed the proposed γ-hydroxy-γ-lactone motif. Finally, in the NOESY spectrum an exchange crosspeak between H-11 and an aldehyde proton at δ_H_ 9.75 ppm confirmed the keto-enol equilibrium suggested for compound **1**. This equilibrium was also observed for compound **2**. A correlation between H-11 and Me-13 (δ_H_ 0.80 *s*) revealed the α-configuration of the hydroxy group at C-11. Therefore, the structure of **1**, named ugandenial A, was established as depicted in Figure 1.

Compounds **2**-**9** were identified as 11α-hydroxycinnamosmolide (**2**) [9], warburganal (**3**), mukaadial (**5**), 9α-hydroxycinnamolide (**7**) [10], polygodial (**4**) [11], dendocarbins A, L and M (**6**, **8** and **9**, respectively) [12] by comparison of spectroscopic data with published values (UV, NMR and MS). Compounds **8** and **9** were isolated as a mixture in a ratio of approximately 1:1.

Compounds **1**-**9** were subjected to a cytotoxic assay against KB cancer cell line. Only compounds **3**-**5** showed any cytotoxic activities, with IC_50_ values of 0.3, 1.0, and 5.3 µM, respectively. The other compounds were inactive at concentrations up to 10 µM. These results confirm that the presence of the dialdehyde function is essential for a strong cytotoxicity [13]. Taxotere was used for a control experiment.

## Experimental 

### General

Optical rotations were measured at 25 °C on a JASCO P1010 polarimeter. IR spectra were measured on a Nicolet FTIR 205 spectrophotometer. ESIMS spectra were obtained on a Navigator Mass Thermoquest. HRESIMS were obtained on a MALDI-TOF spectrometer (Voyager-De STR; Perseptive Biosystems). NMR spectra were recorded on a Bruker spectrometer (500 MHz for ^1^H, 125 MHz for ^13^C and 2D NMR) at 25 °C and/or (600 MHz for ^1^H, 150 MHz for ^13^C and 2D NMR) at 190 K (**1** and **2**), using TMS as an internal standard. Chemical shifts were in ppm, and coupling constants *J* in Hz. HPLC was performed using a Waters Autopurification system equipped with a UV-vis diode array detector (190-600 nm) and a Pl-ELS 1000 ELSD detector (Polymer Laboratory). Precoated silica gel plates (Merck) were used for TLC. Detection was done by spraying plates with 5% anisaldehyde-sulfuric acid, followed by heating. 

### Plant material

Bark of *Warburgia ugandensis* were collected in March 2007 in the high altitude scrubland in Kibale National Park, western Uganda, by one of us (J.K.). A voucher specimen (UFC-0078) is stored in the herbarium of Museum National d'Histoire Naturelle in Paris.

### Cytotoxicity activity assay

The human tumor cell line KB (mouth epidermoid carcinoma) was originally obtained from the ATCC. The cytotoxicity assays were performed according to a published procedure [14].

### Extraction and isolation

The powdered air-dried bark of *Warburgia ugandensis* (200 g) was extracted with EtOAc (3 x 1 L) at room temperature to afford an EtOAc extract (3.06 g), which displayed a significant inhibitory activity on KB cells (99 % and 64% inhibition at 10 and 1 μg/mL, respectively). The EtOAc extract (3.0 g) was subjected to flash column chromatography on silica gel eluting with *n*-heptane-CH_2_Cl_2_ (10%-100%) and CH_2_Cl_2_-MeOH (0%-50%) to give thirteen fractions (F1 to F13). Fractions 7, 8, 10 and 11 were found to be the most cytotoxic (>90% at 10 μg/mL). A semipreparative HPLC using a Kromasil C-18 column (250 x10 μm I.D., 5 μm) with a mobile phase consisting of acetonitrile-water + 0.1% formic acid (70:30) at 4.7 mL/min afforded **3** (7.2 mg, t_R_ 7.1 min) and **4** (47.6 mg, t_R_ 9.8 min) from F7 and **3** (230.8 mg) from F8, respectively. Fractions 10, 11 and 12 were separately subjected to semi-preparative HPLC using a Symmetryshield RP-18 column (150 x19 mm I.D., 5 μm) with a mobile phase consisting of acetonitrile-water + 0.1% formic acid (40:60) at 17 mL/min to yield **6** (1.9 mg, t_R_ 19.8 min), and **7** (17.2 mg, t_R_ 14.4 min) from F10, and **5** (3.2 mg, t_R_ 3.7 min) from F11. Additional prep. TLC using precoated silica gel plates eluted with *n*-heptane-acetone (6:4) afforded **8**+**9** (4.8 mg, R_f_ 0.64), **1** (14 mg, R_f_ 0.67) and **2** (3.2 mg, R_f_ 0.55) from F12.

### Ugandenial A *(**1**)*

Yellow amorphous powder. M.p. 129 °C. [α]_D_ – 28.3 (*c* = 1.8, MeOH). IR ν_max_ cm^-1^: 3417, 1745, 1159; ^1^H- and ^13^C-NMR spectral data: see Table 1. ESIMS *m/z*: 289 [M+Na]^+^. HRESIMS *m/z*: 289.1427 [M+Na]^-^C_15_H_22_O_4_Na (calc. 289.1416)4.

## Conclusions

A chemical investigation of *Warburgia ugandensis* bark was carried out in the framework of a global investigation on the feeding behavior of chimpanzees in Kibale National Park in Uganda. This study showed that the bark contained γ-lactone sesquiterpenoids, of which ugandenial A (**1**) is characterized for the first time, and cytotoxic dialdehyde drimane-type compounds. The presence of dendocarbins A, L and M (**6**, **8** and **9**, respectively) was not previously reported in *W. ugandensis*.

## Figures and Tables

**Table 1:** ^13^C- and ^1^H-NMR spectral data of compounds **1** (δ in ppm, *J* in Hz).

Position	δ_C_	1 ^a^
		δ_H_ (*J* in Hz)
1	30.3	1.62 (*dd,* 11.8, 11.8) 1.20 (*m*)
2	17.5	1.46 (*ddd*, 11.8, 11.8, 11.8) 1.37 (*m*)
3	41.1	1.35 (*m*) 1.14 (*dd,* 11.8, 11.8)
4	33.0	-
5	41.1	1.79 (*dd*, 10.7, 5.0)
6-β 6-α	25.2	2.07 (*dd*, 19.3, 10.7 Hz) 2.32 (*dd*, 19.3, 5.0 Hz)
7	143.3	6.98 (*brs*)
8	129.2	-
9	75.7	-
10	39.2	-
11	98.5	5.66 (*d,* 10 Hz)
12	168.7	-
13	16.1	0.80 (*s*)
14	33.0	0.85 (*s*)
15	21.3	0.87 (*s*)
9-OH		5.00
11-OH		5.90

^a 13^C- (150 MHz) and ^1^H- (600 MHz) in CD_2_Cl_2_ at 190 K.
